# Meet the Editorial Team. An interview with Amitabha Bandyopadhyay, Assistant Editor, Indian Institute of Technology Kanpur, India

**DOI:** 10.1002/dvdy.70137

**Published:** 2026-05-01

**Authors:** Paul Trevorrow, Amitabha Bandyopadhyay

**Affiliations:** ^1^ John Wiley and Sons, Ltd. Bognor Regis West Sussex UK; ^2^ Indian Institute of Technology Kanpur Kanpur India

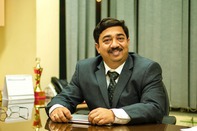

Amitabha Bandyopadhyay is a professor in the department of Biological Sciences and Bioengineering of the Indian Institute of Technology Kanpur. He Chairs the R&D Taskforce of the Gangwal School of Medical Sciences and Technology of IIT Kanpur. Amitabha conducted his doctoral research at Albert Einstein College of Medicine in New York. Upon receiving his PhD in 2002, he joined Harvard Medical School for his post‐doctoral research. In his independent laboratory, he studies the molecular regulation of limb skeleton development, specifically the development of joint cartilage and osteoarthritis, a pathology of skeletal joints. Since July 2018 to August 2022, Amitabha served as the Professor‐in‐charge of Innovation and Incubation and ran the Technology Business Incubator of IIT Kanpur. From January 2021, Amitabha is a member of the National Startup Advisory Council. He is also a member of the Committee for the National Innovation and Startup Policy 2024. He has co‐authored a book titled, “The Ventilator Project: How the IIT Kanpur Consortium Built a World‐class Product during India's Covid‐19 Lockdown” chronicling the extraordinary journey of a group of young IIT Kanpur alumni developing an ICU ventilator in 90 days. Amitabha is a member of several national committees advising/overseeing the startup ecosystem of India, particularly the Healthcare startups.

## 1 CAN YOU TELL US ABOUT YOUR RESEARCH JOURNEY?

My father was an agricultural scientist—a traditional plant breeder—and his work had a big influence on me. India has a wide range of geographic environments, including coastal estuaries where seawater moves inland, making rice cultivation very difficult. My father developed rice varieties that could thrive in those challenging conditions. He crossed low‐yielding wild varieties adapted to saline, flood‐prone areas with high‐yielding varieties from the northern plains, creating rice plants that could grow as water levels rose—up to a foot—and were both salt‐ and flood‐resistant.

He used to show me his research data when I was in grades 8 and 9, and I found it fascinating. From quite early on, I knew I wanted to go into research.

At school my favorite subject was chemistry, whereas I really disliked biology, largely because the Indian biology textbooks—still true today—tend to encourage rote learning. I then went on to study at Presidency College, Calcutta, one of India's most prestigious and historically significant institutions, founded in 1817. Many Indian Nobel laureates have links to the college. I enrolled in the chemistry program, which was a three‐year BSc similar to the UK system.

In the final year, we had to choose a special subject. My mathematics was frankly terrible, so I avoided industrial chemistry and chose biochemistry instead. I bought myself a copy of Stryer's *Biochemistry*, and I must say, Paul, that book completely changed my outlook. I fell in love with biochemistry almost immediately. I went on to do a master's degree in biochemistry in India, and then moved to the Albert Einstein College of Medicine in New York for my PhD, where I focused on molecular biology—specifically protein synthesis, one of the most fundamental cellular processes.

Although my work used biochemical and genetic approaches, my PhD department was Developmental and Molecular Biology, so I was exposed to a lot of inspiring developmental biology seminars, as you typically are in graduate school. That exposure convinced me to move into developmental biology.

Developmental biology relies heavily on embryology and microscopy, and I actually saw my first microscope only when I began my postdoctoral work. I joined Cliff Tabin's laboratory at Harvard Medical School. He is one of the leading developmental biologists of the modern era and is well known for discovering Sonic Hedgehog and elucidating limb patterning.

For the first 2 years in his lab, whenever I was given a research question, I instinctively approached it like a biochemist—and I was invariably wrong. Cliff would remind me, “Amitabha, in developmental biology, it's all about location, location, location.” It took me about 2 years to relearn the philosophy of the field, but by my third and fourth years, things started to click. I could design my own experiments and had a successful postdoc.

When I started my own lab, I initially continued the line of work I had begun in Cliff's group, but gradually I developed an approach that blended philosophies of biochemistry with embryology. My strength lies in understanding transcriptional regulation, so my work focuses on transcriptional control in development, especially in pattern formation and differentiation.

Between 2015 and 2018, my lab published two papers on how cartilage in the developing limb is converted into bone. In the embryo, the appendicular skeleton—the bones of the arms and legs—begins entirely as cartilage. Almost all of it transforms into bone, except for the articular cartilage at the ends. We identified both the molecular and cellular mechanisms behind this transformation.

This led us to a new insight: in osteoarthritis, cartilage is again converted to bone, which is one of the hallmarks of the disease. That raised an intriguing possibility—could osteoarthritis represent, in adults, a reactivation of developmental processes? So in 2018, we established a new research stream focused on adult cartilage maintenance and homeostasis, using principles of developmental biology.

## 2 WHAT IS YOUR CURRENT RESEARCH ABOUT?

### 2.1 At the moment, my lab is focused on three main areas of research

1. Limb skeletal branching and segmentation

If you look at your arm or leg, you'll notice there is one bone proximally, two in the middle, and then five in the hand or foot. What's remarkable is that these are not formed as independent elements—they arise through branching of an original skeletal structure. Branching is well studied in systems like the lung, kidney, and the Drosophila trachea, but there is essentially no literature on the molecular regulation of branching within the skeleton.

This work actually began when I started thinking about another problem. Human height varies, but the ratio between different limb segments—technically, the stylopod and zeugopod—remains strikingly constant. If someone has even a slightly altered ratio, you immediately notice it in a crowd. Despite this invariance, the elements don't form independently; a cartilage template forms first and then segments. I wanted to understand what determines the precise location of that segmentation.

To make a long story short, we drew inspiration from somite segmentation. Olivier Pourquié's work has shown that in somite formation, reciprocal gradients of retinoic acid and FGF determine when and where segmentation occurs. Because these same signaling pathways operate in analogous positions in the limb bud, we hypothesized a similar mechanism. We found that RA–FGF reciprocal gradients indeed specify segmentation sites in the limb.

For these experiments, we used FGF8. During the COVID period, we ran out of FGF8 protein, so my student used FGF4 instead—assuming functional redundancy, as consistently stated in the literature. To our surprise, segmentation remained unchanged, but an extra skeletal branch formed. That observation opened an entirely new direction. We are now investigating the molecular mechanism underlying limb skeletal branching, and we've already made substantial progress.

2 Coordinated development of muscle, tendon, and cartilage

When you move your skeleton, muscles generate force, but that force is transmitted via tendons to the bones. Anatomically, the transition from muscle to tendon to cartilage is seamless, which makes it unlikely that these tissues develop completely independently and then simply connect themselves. There must be a co‐developmental program.

In the axial skeleton, Cliff Tabin's lab showed long ago that the somite contains two key domains: the myotome, which forms muscle, and the sclerotome, which forms cartilage. These interact to produce the syndetome—the progenitor of tendon—ensuring natural, induced connectivity.

The limb, however, is different because limb muscles do not form in situ; they migrate in from the somites. That raises the question of how the muscle‐tendon–cartilage linkage is established in the limb. We have made a breakthrough here: we have identified a gene expressed specifically at the tendon–cartilage attachment point. We believe we've also located the origins of limb tendons and are now characterizing the mechanisms involved in establishing these connections.

3 Understanding osteoarthritis through developmental biology

On the translational side, we study osteoarthritis. Age is the strongest risk factor—around 50% of people over 65 develop osteoarthritis. Obesity is the next major factor. People often assume this is due to increased mechanical load, but some non–load‐bearing joints also develop osteoarthritis in obese individuals. That suggests weight alone cannot be the whole story.

Obesity is associated with chronic inflammation, with macrophages playing a central role. From a developmental biology perspective, cartilage must convert into bone for osteoarthritis to occur, and BMP signaling is essential for that process. We are therefore testing the hypothesis that macrophages act as a source of BMP ligands in obesity, triggering pathological cartilage‐to‐bone conversion.

We are also studying why osteoarthritis incidence diverges sharply between men and women after menopause. Until around age 55, men are more susceptible to osteoarthritis than women; after menopause, the rate in women rises dramatically. Surprisingly, it has not been examined thoroughly whether menopause itself directly affects cartilage health. Our findings indicate that estrogen has a cartilage‐protective role. While systemic hormone replacement therapy has well‐known risks, local delivery of estrogen agonists into the joint could offer protection with minimal side effects. These drugs already exist, so translation would mainly involve adjusting dosage and delivery.

So those are the three areas my lab is actively exploring: limb skeletal branching, coordinated development of muscle–tendon–cartilage connectivity, and osteoarthritis from a developmental perspective.

## 3 WHERE DO YOU SEE THE FUTURE OF THE FIELD?

This is a fairly standard frustration I have with the developmental biology community—and I say this as someone who is very much part of that community. Stem cell biology, for example, grew directly out of developmental biology. Many of the leading figures in stem cell science today started their careers as developmental biologists. Yet, at some point, the field stopped viewing that work as developmental biology and allowed it to spin out into its own discipline. We all know what has happened since—stem cell biology has become enormously influential.

Similarly, I believe the future of the field should involve what I would call the *developmental origin of disease*. Many chronic diseases, in my view, should be reinterpreted through developmental biology principles—processes like differentiation, proliferation, and lineage decisions going wrong.

Osteoarthritis, for me, is one such example. I have published work using developmental biology frameworks to investigate mechanisms of bone homeostasis in adults. But developmental biology journals rarely encourage this type of interpretation. I have had papers desk‐rejected with the comment that they “do not offer anything new to developmental biology”, which, strictly speaking, is true—you don't learn a new developmental mechanism. What you *do* learn is how developmental biology continues to operate in adult physiology and pathology. I think that perspective is essential if the field is to remain relevant.

To be clear, I have nothing against fundamental research. I am a fundamental developmental biologist myself, and there are still many unknowns to uncover. But adding an applied dimension would broaden and strengthen the field rather than weaken it.

Take tissue engineering as an example. Tissue engineers try to generate adult tissue types in vitro, often using stem cells as the starting material. But the starting material alone is not enough; the cells require the right developmental cues. Many tissue engineers do not necessarily have developmental biology training, but if we collaborate—if we build a discipline of *developmental biology‐inspired tissue engineering*—I think the field could move forward much more effectively.

## 4 WHAT ADVICE WOULD YOU GIVE YOUR YOUNGER SELF?

Career‐wise, I'm quite content with what I've been able to do. Perhaps I'm even *too* content. I've certainly made plenty of mistakes along the way, and I'll share one example.

My PhD supervisor was an old‐fashioned biochemist and a master of protein purification. I was part of the last generation still cloning genes for the first time, before the human genome was published. I was working on a translation initiation factor called eIF3, which in mammals has 10 subunits. Translation is usually very conserved between yeast and mammals, but in this case yeast—*S. cerevisiae*—had only 5 of the 10 genes. That meant genetics was limited, because half the subunits simply weren't there, and biochemistry alone couldn't tell us what individual subunits did.

So in 1996, I proposed using the baculovirus expression system to reconstitute the full 10‐subunit complex and then remove one subunit at a time to see which functions were affected. It sounded reasonable to me. My PI warned me not to do it. I disagreed. He finally said, “Go ahead. But if it doesn't work, don't come back and bang your head against the wall.”

I went ahead—and he was absolutely right. After 4 years, I managed to purify the full complex, and it behaved exactly like the native protein, except for one crucial detail: it had no activity.

Now, if you ask me whether I would encourage one of my own PhD students to take on a risky, challenging project like that, the answer is 100% yes. If it works, it's wonderful. If it doesn't, you still learn an enormous amount. A PhD is the phase where you learn how to think. The techniques and data matter far less than the development of your thought process.

Those three and a half “failed” years shaped the way I think as a scientist.

Reflecting on your question—nobody has ever asked me this before—I think I would make the same choices again. Perhaps I would just study a bit more chemistry during my undergraduate years.

## 5 WHAT ARE YOUR FAVORITE PASTIMES OUTSIDE OF RESEARCH?

If I can find the time, I absolutely love to travel. I've traveled a fair amount already, and it's something I'd happily do much more of. When traveling isn't possible and I'm at home, I enjoy watching films. I'm a little sad to admit that I used to be an avid reader, but my attention span for books has definitely suffered because of work.

So, in order of preference, it would be: traveling, watching films, and then books.

My ideal destination is somewhere by the sea, especially places with coral reefs where you can snorkel or dive. But my wife doesn't like water, so when that's not an option, I happily settle for the mountains—of which there is no shortage in India. Hills, mountains, different ranges—I love exploring them all.

## Data Availability

Data sharing not applicable to this article as no datasets were generated or analysed during the current study.

